# Mental health and psychosocial support in response to onset of the COVID-19 pandemic: Emotional and Stress Management Intervention in Liberia, Sierra Leone, and Ivory Coast

**DOI:** 10.7189/jogh.13.06010

**Published:** 2023-07-14

**Authors:** Anna Garriott, Xuan Phan, Karine Le Roch, Fatmata Massah Bintu, Satta Paasewe Darciba, Placide Koleti, Sarah M Murray

**Affiliations:** 1 Department of Mental Health, Johns Hopkins Bloomberg School of Public Health, Baltimore, Maryland, USA; 2 Action contre la Faim, Paris, France; 3 Action Against Hunger, Freetown, Sierra Leone; 4 Action Against Hunger, Monrovia, Liberia; 5 Action contre la Faim, Abidjan, Ivory Coast

## Abstract

**Background:**

In response to the psychological distress experienced by people affected by the onset of the COVID-19 pandemic, Action Against Hunger (Action contre la Faim, ACF) developed and implemented the Emotional and Stress Management Intervention (ESMI) in Liberia, Sierra Leone, and Ivory Coast. ESMI is a person-to-person two-session, non-specialized, mental health and psychosocial support intervention for adults and adolescents in the general population based in problem solving therapy and principles of emotional regulation.

**Methods:**

Using de-identified programmatic data for each country, we conducted paired *t*-tests to assess whether adults and adolescents who received ESMI experienced changes in reported psychological distress and perceived social support following the intervention. We also performed pairwise correlations to test whether there were associations between changes in distress and social support over the course of participation in ESMI. Descriptive analyses were performed for presenting problems and coping strategies reported during the sessions.

**Results:**

Mean scores for psychological distress at baseline and follow-up were significantly different in all countries: Sierra Leone (mean (m) = -6.11; 95% confidence interval (CI) = -6.25 to -5.96); Ivory Coast (m = -3.21; 95% CI = -3.33 to -3.10); and Liberia (m = -2.86; 95% CI = -3.15 to -2.56). Changes in perceived social support were also statistically significant for Sierra Leone (m = 6.87; 95% CI = 6.72-7.02); Ivory Coast (m = 3.12; 95% CI = 2.97-3.27); and Liberia (m = 1.13; 95% CI = 1.00-1.27). Correlations (r) between changes in distress and changes in social support varied by country, and ranged from negative in Liberia, (r = -0.88, *P* = 0.001), to positive in Ivory Coast (r = 55, *P* = 0.001), and null in Sierra Leone (r = -0.07, *P* = 0.11).

**Conclusions:**

Our findings show changes in psychological distress and perceived social support for participants who completed two sessions of ESMI, suggesting a potential benefit of ESMI as a person-to-person mental health and psychosocial support for individuals in distress affected by a pandemic. A future randomized controlled trial with a focus on key implementation factors (i.e., pre-testing visual analogue scales, treatment fidelity, and comparison of in-person vs remote) is recommended as next steps in research.

The COVID-19 pandemic and associated response brought unprecedented psychological distress across the world. Physical and social isolation, disruptions to daily activities (e.g. school, work, and childcare), loss of livelihoods, social injustice, fear of infection, stigma, and grief impacted populations across the globe [1,2]. A Lancet systematic review [3] estimated that the global prevalence of depression and anxiety increased by 27.6% and 25.6% respectively due to the COVID-19 pandemic and its socio-economic impact. In addition, women and younger age groups were identified as being at greater risk for psychological distress during the pandemic as compared to men and older adults, respectively [3].

While the literature has broadly demonstrated critical mental health impacts of the pandemic, few studies have assessed change in mental health related to COVID-19 within African countries. Studies that do exist suggest heterogeneous experiences within the region that are context specific [2,4-6]. In the first year of the pandemic, the African region did not experience as high rates of SARS-CoV-2 as other world regions or as anticipated by experts [7-9]. However, research that does exist on the effects of COVID-19 lockdowns in African countries has found increases in psychological distress expressed as fear, worry, boredom, frustration, anger, and anxiety, with unemployment and living in large households associated with greater emotional distress [4]. Reports [10,11] have also suggested increased domestic violence associated with lockdowns in sub-Saharan Africa (SSA), but the scarcity of publications make it difficult to fully understand the changes in prevalence and the extent of the impact of the pandemic on domestic and gender-based violence.

Only a small number of Mental Health and Psychosocial Support (MHPSS) studies on outbreaks have focused on person-centred psychosocial or mental health support interventions for children, adolescents, and adults in the general population [12]; yet, programmes focused on non-specialized MHPSS services for individuals and groups and strengthening community supports are commonly implemented in humanitarian response [13-15]. In conducting a systematic review of psychosocial interventions in humanitarian settings within low- and middle-income countries, Haroz et al. [15] discovered that most of the evaluation studies that have been done on MHPSS interventions took place in sub-Saharan Africa, typically in response to conflict or the human immunodeficiency virus (HIV) epidemic, and were delivered within health or protection systems to reduce symptoms of psychological distress and increase well-being among children and / or adults. The majority of these studies showed some benefit – i.e., lowering symptoms of distress or improving a measure of quality of life (e.g., self-esteem, functioning, reduction of alcohol use), though the vast majority of these evaluations were observational or quasi-experimental.

In response to the mental health impacts of the COVID-19 pandemic and following the guidelines of the Inter-Agency Standing Committee (IASC) Reference Group for Mental Health and Psychosocial Support (IASC MHPSS RG) [16], Action Against Hunger (Action contre la Faim, ACF), an international non-government organisation (INGO), implemented a brief Emotional and Stress Management Intervention (ESMI) from May to December 2020 in Sierra Leone, Ivory Coast, and Liberia. ACF has worked in these countries for the past three decades implementing programmes in nutrition and health; water, sanitation, and hygiene (WASH); food security and livelihoods (FSL); and MHPSS. The main goal of ESMI was to reduce symptoms of emotional distress and increase perceived social support through problem solving therapy and relaxation exercises provided to adults and adolescents in the general population who were experiencing increased levels of distress due to being directly or indirectly affected by COVID-19.

The primary aim of this study is to evaluate whether individuals who received ESMI experienced changes in psychological distress and social support following the intervention and the association between change in psychological distress and change in perceived social support for each country. The secondary aim is to provide descriptive analysis of the programme participants in reported symptoms of distress and identified problems at baseline and corresponding coping strategies noted in ESMI sessions. Lastly, this study aimed to explore the association of change in psychological distress and change in social support with demographic characteristics (e.g., sex, age, household structure).

## METHODS

This study is a non-randomized observational programme evaluation with two time points – pre- and post-intervention. The study consisted of secondary analysis of de-identified data collected via routine monitoring and evaluation activities by ACF for their ESMI programmes in 2020. Given the use of only de-identified secondary programmatic data, the Johns Hopkins Institutional Review Board issued a “Not Human Subjects Research” determination for this study in accordance with the Department of Health and Human Services regulation 45 CFR 46.102 [17]. Meetings with each of the ACF MHPSS country teams were held to further understand the implementation of ESMI in their respective context.

### Study population and design

Adults and youth (12-17 years old) who participated in ESMI were living in the major cities of Liberia, Ivory Coast, and Sierra Leone. In each country, ACF targeted areas with vulnerable communities experiencing a relatively high prevalence of COVID-19 within the existing geographical coverage of ongoing ACF programmes. ESMI was also implemented in Madagascar in 2020, but due to the high percentage of loss to follow-up, the data set was removed from this study. There were some children (7-11 years old) who received ESMI in Ivory Coast and Sierra Leone but due to the small number, their data were also removed from the final data analyses.

#### Liberia

In Liberia, adults living in the capital city of Monrovia were enrolled in ESMI during July 2020. The intervention was originally planned to be implemented within health facilities, but because of a health personnel strike, it was moved to the community. During community health sensitizations, psychosocial officers trained in Psychological First Aid (PFA), from Community Health Initiative (CHI), a local health care and social services organisation, identified and enrolled adults presenting with signs of psychological distress. The first ESMI sessions were held in person and the second sessions were conducted over the phone.

#### Ivory Coast

In Ivory Coast, adults and youth living in Abidjan were enrolled in ESMI between May and December 2020. ACF partnered with the Ministry of Health and Public Hygiene to deliver ESMI in person with social workers from the National Health Program of School and University – Adolescent and Youth Health at health centres based at schools or other local community centres. Participants were referred to ESMI by staff at health care centres, community workers, leaders in the community, and by word-of-mouth.

#### Sierra Leone

In Sierra Leone, adults and youth in the capital of Freetown were enrolled in ESMI between June 2020 and September 2020. The MHPSS team collaborated with the district health medical team (DHMT) to identify individuals impacted by COVID-19 who presented with signs of psychological distress. Psychosocial officers, trained in PFA, from Community Association for Psychosocial Services (CAPS), a local mental health organisation, held psychoeducation sessions in the community and referred adults and youth who had signs and reported symptoms of psychological distress to ESMI. Community health workers from DHMT also received MHPSS training and referred people to ESMI. Persons who were staying in quarantine homes were provided sessions by phone and persons who were discharged from quarantine had sessions in person and then over the phone. Some households had multiple family members who received ESMI. For individuals with higher levels of distress at the follow-up session, at the discretion of the psychosocial worker, additional phone calls were provided for further support.

### Emotional and Stress Management Intervention (ESMI)

ESMI was designed by ACF as a person-to-person psychosocial support intervention to reduce signs of emotional distress and improve well-being among people affected by the COVID-19 pandemic. It consists of two sessions based on a problem solving approach and emotional and stress management techniques. ESMI was created to be implemented in person or remotely depending on the severity of the pandemic, needs of the local context, and current government restrictions.

At baseline, the psychosocial worker collected demographic information and asked open-ended questions to each participant about the current problems they were experiencing and their present symptoms of distress. A list of symptoms and problems were given to the psychosocial workers to check off based on the responses provided by each person. Psychological distress and social support were measured at baseline and after the end of the second session.

### Measures

ACF used culturally relevant visual analogue scales (VAS) to measure psychological distress and social support among populations with low levels of literacy that have been widely used in ACF MHPSS programming with acceptability by participants. However, these tools have yet to be formally tested for validity in each of the countries where they were implemented. Mental health studies validating VAS measurements have shown the usefulness and validity of visual aids for participants with low levels of literacy [18] and the importance of qualitative and quantitative research methods for determining the cultural relevance and validity of the tool for its intended purpose [19].

Psychological distress was the main outcome of interest, measured by a self-reported 10-point suffering scale (0 = no distress to 10 = severe distress). On the scale, there were four images of a person carrying a small, medium, large, and exceptionally large boulder on their back with numbers below along a continuum (0, 2, 4, 6, 8, 10). The psychosocial worker asked each person to rate their present feelings of distress based on their current problems and challenges in their life. The ESMI instructions recommended that psychosocial workers gave a verbal description of the levels: 0 to 2 = “I feel I can deal with my distress”; 3 to 5 = “I feel it is difficult to deal with my distress and my daily life is affected”; 6 to 7 = “I feel my daily life is very affected by my distress”; 8 to 10 = “I feel my daily life is overwhelmed by my distress”.

Social support was measured with a 10-point perceived social support scale (0 = no support to 10 = well supported) with five images of a person sitting down alone, someone talking to the person sitting down, the person sitting down talking to the person standing up, someone wrapping their arm around the person as they are both talking, and the person standing up and walking with the support of the other person. Psychosocial workers were trained to ask participants, “How much do you feel supported by the people in your life (i.e., family, friends, others) on a scale of from 0 to 10?”

### Data analyses

All analyses were performed separately for each country’s data set using Stata/BE 17.0 [20]. Exploratory data analyses were performed for the variables of interest including, psychological distress, social support, symptoms of distress, identified problems, coping strategies, and demographic characteristics. We used Shapiro and Wilk’s normality test for the continuous data in each country’s data set [21] and pre-specified statistical significance to a *P*-value of <0.05. Paired *t*-tests were used to determine the differences in the mean scores for emotional distress and social support at baseline and follow-up for each country. Pairwise correlations were computed for changes in distress and changes in social support. Bivariate analyses and linear regressions were performed with the primary outcome variables of change in distress and change in social support and covariates of age (continuous and categorical), sex, and household status. Bootstrap estimation [22] with replications of 1000 were used where the outcome was not normally distributed.

## RESULTS

### Demographic characteristics

The ESMI programme was provided to 312 adults in Liberia, 616 adults and youth in Ivory Coast, and 484 adults and youth in Sierra Leone (**Table 1**). The mean (m) age of adults in Liberia was 38.9 (standard deviation (SD) = 11.1) with an age range of 19 to 68 years. In Ivory Coast, the mean age was 28.6 (SD = 11.8) with an age range of 12 to 72 years; and in Sierra Leone, the mean age was 30.0 (SD = 14.2) with an age range of 12 to 80 years. Across all three country sites, more women and adolescent girls (Liberia: 51.9%, Ivory Coast: 64.0% and Sierra Leone: 56.0%) received ESMI and most lived with family members or others (Liberia: n = 244 (78.20%), Ivory Coast: n = 451 (73.21%), Sierra Leone: n = 413 (85.33%). Out of the 1412 participants assessed at baseline, 1350 (95.6% of participants), reported their level of distress at follow-up.

**Table 1:** Emotional and Stress Management Intervention (ESMI) participant characteristics by country at baseline

	Liberia	Ivory Coast	Sierra Leone
**Observations, n**	312	616	484
**Characteristics**			
Sex, n (%)			
*Male*	150 (48.08)	222 (36.04)	213 (44.01)
*Female*	162 (51.92)	394 (63.96)	271 (55.99)
Age, years, m (SD)	38.92 (11.09)	28.58 (11.82)	29.98 (14.15)
Age categories, n (%)			
*12-17*	N / A	108 (17.53)	103 (21.28)
*18-25*	33 (10.58)	188 (30.52)	132 (27.27)
*26-59*	267 (85.58)	310 (50.32)	232 (47.93)
*60+*	12 (3.85)	10 (1.62)	17 (3.51)
Household structure, n (%)			
*Live alone*	68 (21.79)	165 (26.87)	71 (14.67)
*Live alone with children*	78 (25.00)	n/a	112 (23.14)
*Live with family*	166 (53.21)	446 (72.40)	299 (61.78)
*Other / unknown*	N / A	5 (0.01)	2 (0.00)

m – mean, SD – standard deviation, N / A – not applicable

### Symptoms of distress

Across Liberia, Sierra Leone, and Ivory Coast anxiety and sleeping disturbances were symptoms most shared by participants. In Liberia, 99.7% of participants reported anxiety and 87.5% identified problems with sleep. In Sierra Leone, 42.6% reported anxiety and 25.6% sleep disturbances. In Ivory Coast, only one symptom of distress was recorded per person with 19.5% reporting anxiety and 33.8% sleep disturbances. Participants in Sierra Leone had the greatest number of symptoms of distress reported at baseline: 46.9% sadness or crying, 40.9% thinking too much, 35.7% anger, 33.3% difficulty concentrating, 27.3% traumatic stress, 27.1% behavioural problems, 26.2% isolation, 24.6% eating issues, 15.1% irritability, 11.4% fatigue, and 5.8% addictive behaviours. In Ivory Coast, other reported symptoms of distress, apart from anxiety and sleep disturbances, included difficulty concentrating (13.7%), sadness and crying (9.6%), loss of pleasure (6.7%), isolation (3.4%), aggression or violence (3.2%), anger (3.2%), rumination (1.6%), and irritability (1.1%).

### Problems identified

Fear of infection ranked among the most prevalent problems identified by participants in all three countries: 99.7% in Liberia, 59.4% in Ivory Coast, and 61.2% in Sierra Leone. Stigma and socio-economic hardships were also frequently reported problems among participants. In Sierra Leone, 75.4% of participants reported stigma related to COVID-19 with 66.9% of participants identifying socio-economic difficulties. In Ivory Coast, 29.2% of participants mentioned socio-economic problems, the second most frequently identified problem after fear of being infected. Other types of problems were less frequently reported (<3%) and varied by participants, i.e., fear of isolation, fear of losing someone close, and fear of separation.

### Coping strategies

The most frequently reported resources to reduce distress in Sierra Leone were social support (57.4%), leisure activities (56.4%), physical and outdoor activities (26.4%), spiritual activities (16.1%), and counselling (3.9%). In Liberia, participants most often identified self-care strategies (42.0%), counselling (20.5%), provision of information (13.5%), leisure activities (12.5%), spiritual activities (8.0%), and physical activities (6.1%) as methods for reducing distress. Psychological workers in Ivory Coast did not record coping strategies to reduce distress but documented strategies for managing problems that participants identified, which included implementing personal safety measures against COVID-19 (e.g. hand washing and social distancing) (30.0%), receiving accurate information about COVID-19 (13.8%), working or studying (15.3%), participating in physical activities (8.8%), and engaging in spiritual activities (1.8%).

### Changes in emotional distress and social support

**Figure 1:**
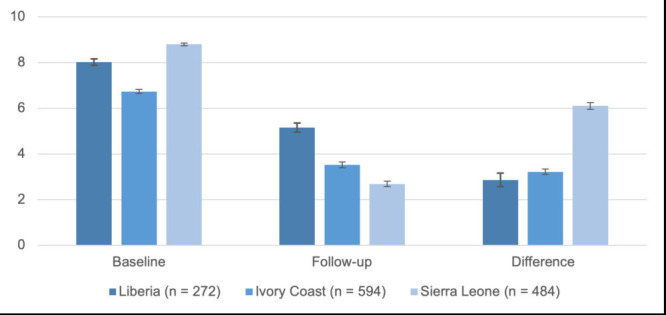
Mean change in emotional distress between baseline and follow-up by country. Mean scores (95% confidence intervals in brackets) reported for emotional distress by country at baseline and follow-up for participants who completed two sessions; mean (95% confidence intervals in brackets) differences calculated from paired *t* tests. Distress scale: 0 = manageable level of distress to 10 = overwhelming level of distress. Mean differences from paired *t* tests for each country were significant (*P* < 0.001). Difference reported in absolute terms.

Displayed in **Figure 1**, mean levels of distress reported by participants in all three countries decreased between baseline and follow-up of ESMI. In Sierra Leone, participants had higher average level of distress at baseline (m = 8.79, SD = 0.65) compared to Liberia (m = 8.02, SD = 1.18) and Ivory Coast (m = 6.74, SD = 1.10). At follow-up, participants in Sierra Leone had the lowest average level of distress (m = 2.69, SD = 1.36) followed by Ivory Coast (m = 3.52, SD = 1.51) and Liberia (m = 5.16, SD = 1.66). These differences were statistically significant across all three countries: Sierra Leone (m = -6.11; 95% CI = -6.25 to -5.96, *t* (483) = -84.18, *P* < 0.001); Ivory Coast (m = -3.21; 95% CI = -3.33 to -3.10, *t* (593) = -54.64, *P* < 0.001); and Liberia (m = -2.86; 95% CI = -3.15 to -2.56, *t* (271) = -19.05, *P* < 0.001).

**Figure 2:**
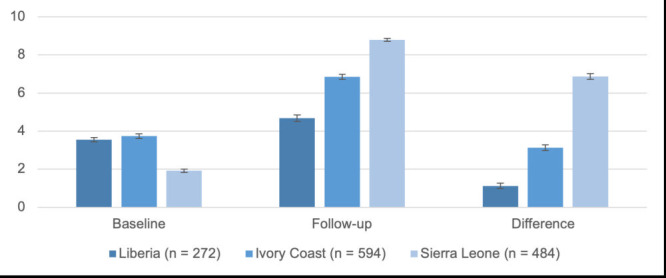
Mean change in social support between baseline and follow-up by country. Social support mean scores (95% confidence intervals in brackets) by country at baseline and follow-up for participants who completed two sessions; mean differences (95% confidence intervals in brackets) calculated from paired *t* tests. Social support scale: 0 = no support to 10 = well-supported. Mean differences from each paired *t* test were significant (*P* < 0.001). Difference reported in absolute terms.

Average scores in social support increased in all sites from baseline to follow-up as shown in **Figure 2**. Sierra Leone had the lowest mean level of social support at baseline (m = 1.92, SD = 1.08) with Liberia (m = 3.55, SD = 0.92) and Ivory Coast (m = 3.74, SD = 1.50) having similar mean scores. At follow-up, Sierra Leone (m = 8.78, SD = 6.87) had the highest level of social support followed by Ivory Coast (m = 6.86, SD = 3.12) and Liberia (m = 4.68, SD = 1.13). The changes in social support were statistically significant for all three countries: Sierra Leone (m = 6.87; 95% CI = 6.72-7.02, *t* (483) = 87.63, *P* < 0.001); Ivory Coast (m = 3.12; 95% CI = 2.97-3.27, *t* (593) = 40.38, *P* < 0.001); and Liberia (m = 1.13; 95% CI = 1.00-1.27, *t* (271) = 16.55, *P* < 0.001).

Changes in distress and changes in social support were negatively correlated in Liberia, r (270) = -0.88, *P* = 0.001. Ivory Coast had a positive correlation between change in distress and change in social support, r (592) = 0.55, *P* = 0.001. In Sierra Leone, change in distress and social support were not statistically significant, r (482) = -0.07, *P* = 0.11.

The results of the linear regressions by country for the outcome variables change in distress (**Table 2**) and social support (**Table 3**) and demographic characteristics included as covariates showed a significant mean difference in change in distress, β = -0.29; 95% CI = -0.57 to -0.01, among participants in Ivory Coast who lived alone compared to living with others, indicating a greater decrease in average change in distress with living alone. Being older in age in Ivory Coast predicted a slightly greater decrease in distress, *β =* -0.01; 95% CI = -0.02-0.00. In Sierra Leone, youth compared to adults on average had a greater increase in change in distress, β = 0.46; 95% CI = 0.02-0.90, and living alone compared to living with others also had a significant mean difference, β = -0.68, 95% CI = -0.95 to -0.40, with living alone on average being associated with a greater decrease in change of distress. There were no statistically significant differences (*P* < 0.05) in change in distress and demographic characteristics for Liberia. Apart from Sierra Leone having a mean difference in age and change in social support, β = 0.01; 95% CI = 0.00-0.02, there were no statistical significant differences in changes in social support and demographic characteristics (**Table 3**).

**Table 2:** Linear regressions for change in distress and association with socio-demographic characteristics by country

	Liberia		Ivory Coast		Sierra Leone	
**Observations, n**	272*		594*		484	
**Variables**	β (95% CI)	*P*-value	β (95% CI)	*P*-value	β (95% CI)	*P*-value
Age	0.01 (-0.02 to 0.03)	0.61	-0.01 (-0.02 to 0.00)†	0.02†	0.00 (-0.01 to 0.01)	0.99
Sex						
*Male*	Ref.		Ref.		Ref.	
*Female*	0.00 (-0.60 to 0.59)	0.99	-0.22 (-0.46 to 0.02)	0.07	0.02 (-0.25 to 0.30)	0.86
Age category						
*Adults (18+)*	N / A		Ref.		Ref.	
*Youth (12-17)*	N / A		-0.03 (-0.37 to 0.31)	0.85	0.46 (0.02-0.90)	0.04
Household status						
*Live with others‡*	Ref.		Ref.		Ref.	
*Live alone*	0.16 (-0.51 to 0.82)	0.65	-0.29 (-0.57 to -0.01)†	0.04	-0.68 (-0.95 to -0.40)†	0.01†
R-squared	0		0.03		0.04	
Wald χ^2^	χ^2^ = 0.47		χ^2^ = 16.67		χ^2^ = 36.61	
*P-*value	0.92		0.01		0.01	

CI – confidence interval, N / A – not applicable or no data

*Participants lost to follow-up included 40 participants in Liberia and 22 participants in Ivory Coast.

†Significant *P*-values (*P* < 0.05).

‡Live with others includes participants who reported living with their children, partner, other family members, or non-family members.

**Table 3:** Linear regressions for change in social support and association with socio-demographic characteristics by country

	Liberia		Ivory Coast		Sierra Leone	
**Observations, n**	272*		594*		484	
**Variables**	β (95% CI)	*P*-value	β (95% CI)	*P*-value	β (95% CI)	*P*-value	
Age	0.00 (-0.02 to 0.01)	0.42	-0.01 (-0.02 to 0.01)	0.51	0.01 (0.00-0.02)†	0.02†	
Sex							
*Male*	Ref.		Ref.		Ref.		
*Female*	-0.04 (-0.32 to 0.23)	0.74	-0.27 (-0.58 to 0.03)	0.08	-0.02 (-0.32 to 0.28)	0.89	
Age category							
*Adults (18+)*	N / A		Ref.		Ref.		
*Youth (12-17)*	N / A		0.18 (-0.30 to 0.66)	0.46	0.44 (-0.03 to 0.91)	0.07	
Household status							
*Live with others‡*	Ref.		Ref.		Ref.		
*Live alone*	0.07 (-0.22 to 0.37)	0.62	-0.03 (-0.41 to 0.35)	0.88	0.18 (-0.19 to 0.56)	0.33	
R-squared	0		0.01		0.01		
Wald χ^2^	χ^2^ = 1.01		χ^2^ = 5.57		χ^2^ = 5.68		
*P-*value	0.80		0.23		0.23		

CI – confidence interval, N / A – not applicable

*Participants lost to follow-up included 40 participants in Liberia and 22 participants in Ivory Coast.

†Significant *P*-values (*P* < 0.05).

‡Live with others includes participants who reported living with their children, partner, other family members, or non-family members.

## DISCUSSION

In this study in three different African countries, we found that individuals who participated in ESMI, a brief problem solving and stress management intervention, experienced reduced psychological distress and improvements in perceived social support. We also found limited evidence of changes in distress associated with participant characteristics and no evidence of changes in social support associated with demographics.

As found in other studies of MHPSS interventions [3,23], more women participated in ESMI across all three countries than men. There were no observable differences by gender in change in distress reported at follow-up among ESMI participants. Langsi et al. [4] in their research on mental health in SSA during the pandemic found that living alone increased the odds of emotional distress. We found at baseline, that there were no differences in distress among those living alone, but there were on average slightly greater reduction in distress levels among participants living alone in Ivory Coast and Sierra Leone. We also found that youth in Sierra Leone had on average an increase in distress. Other research in SSA during the pandemic [3] has shown youth being more at risk for psychological distress and further research is required to understand how ESMI can be adapted as a youth friendly intervention.

On average in all three sites, participants reported higher perceived social support between baseline and follow-up. However, only in Liberia did we find an increase in social support associated with a decrease in distress. Social support has been emphasized in the COVID-19 mental health literature as a promotive factor for well-being [24] and as a mediator in reducing psychological distress [25]. While our study contributes to this body of research, more studies are needed to further understand the cultural and social nuances of the type of social supports that are beneficial during pandemics in the African context.

While we found anxiety and sleep disturbances were most frequently reported for symptoms of distress across the three countries, due to the varying data collection methods by each country it is difficult to make a true comparison of the differences in the number and frequency of symptoms between countries. Fear of infection, stigma, and socio-economic problems were shared identified problems across the three countries. This is not surprising given the limited public health knowledge about the novel coronavirus, their experience of Ebola, and the widespread food and economic insecurities because of the pandemic and related government epidemic control policies [11,26,27]. The type of coping strategies in our study varied by each country with little overlap, except for self-care strategies being identified in all three countries. Future qualitative research on coping strategies in SSA is needed to further our understanding on how and why certain coping strategies may present as risk or protective factors for lowering distress.

Findings from this study should be interpreted with consideration for several limitations. Collecting data consistently across countries as part of routine programme monitoring is challenging due to differences in staff’s education background and technical training, competing demands on staff time, and other cultural and contextual factors in programme implementation related to how, when, where, and who is implementing the programme and collecting the data. Loss to follow-up and missing data are limitations to programmatic evaluations and were the case in Liberia and Ivory Coast. The visual analogue scales (VAS) used in assessment of distress and social support can be an acceptable and accessible means of verification [18,28], yet the subjective nature of the scales makes it difficult for accurate comparisons across cultural contexts and these scales were not specifically validated within each population. Further research is needed to test the unidimensional scales against other validated assessment tools and compare with clinical assessment [29]. The fidelity of using the scales over the phone without a visual aid for participant in quarantine in Sierra Leone may have impacted the quality of the reporting, and further research is needed to assess this and validate the measurements against a clinician’s assessment or other standardized psychometric tools. In Liberia, there appeared to be some questions on the validity in the data collection for problems identified at baseline as almost all participants endorsed the same problems. While there are likely similarities in the collective experience of individuals from the same community during a pandemic, the nuances found at the individual level in the other countries were more varied and raise concern for Liberia’s data. There may be aspects of social support at the individual, household, neighbourhood, community, and country levels that are unmeasured by the data in our analyses and may be a confounder in the relationship between ESMI and the outcome measurements. Most importantly, observational studies without a control group by design do not allow for causal inference, and we expect distress scores to reduce over time due to regression to the mean or the changing situation of the pandemic. However, this observational design with baseline and end-line comparisons is still useful for assessing whether change was observed as intended in the programme design [30]. Further, how that change may have varied among diverse participants can generate information about targeting programmes to those most in need and who stand to most benefit.

ESMI proved to be a flexible, brief intervention that could be adapted to different settings and age groups depending on the country and local context. Further work is needed to design and implement a community-based randomized controlled trial, complete with sample size calculations, for ESMI in humanitarian contexts. In the long-term, we recommend adaptation of the intervention as a child- and youth-friendly support for children and adolescents and exploration of integrating ESMI into other sectors such as health and nutrition. Comparing the effectiveness of remote vs. in-person sessions is another area for future research. From the country-level MHPSS team feedback, we learned that for some participants two sessions were not enough to reduce distress and further sessions were needed. Timing between sessions is another factor to further examine as well as following-up with participants later, such as three or six months after, to see if they are still using the skills learned in the intervention.

## CONCLUSIONS

Given the low frequency of evaluations of psychosocial programming and discussion of implementation of these programmes in the literature, this adequacy evaluation study can provide important contextual understanding of the delivery of a MHPSS intervention for the general population in response to the COVID-19 pandemic. Specifically, this study adds important findings to a growing body of literature on how a brief MHPSS intervention during the onset of a pandemic is associated with reducing distress and increasing social support across a diverse age range and geographic settings. Based on the promising findings of this study, we recommend future efforts to validate the visual analogue scales (VAS) and conduct a community randomized controlled trial for ESMI.

## References

[1] Interagency Standing Committee. IASC Guidance on Basic Psychosocial Skills – A Guide for COVID-19 Responders. Available: https://interagencystandingcommittee.org/iasc-reference-group-mental-health-and-psychosocial-support-emergency-settings/iasc-guidance-basic-psychosocial-skills-guide-covid-19-responders. Accessed: 18 April 2022.

[2] Chiesa V Antony G Wismar M Rechel B COVID-19 pandemic: health impact of staying at home, social distancing and ‘lockdown’ measures—a systematic review of systematic reviews. J. Public Health (Oxf.). 2021;43:e462-81. 10.1093/pubmed/fdab102 33855434 PMC8083256

[3] COVID-19 Mental Disorders Collaborators Global prevalence and burden of depressive and anxiety disorders in 204 countries and territories in 2020 due to the COVID-19 pandemic. Lancet. 2021;398:1700-12. 10.1016/S0140-6736(21)02143-7 34634250 PMC8500697

[4] Langsi R Osuagwu UL Goson PC Abu EK Mashige KP Ekpenyong B Prevalence and Factors Associated with Mental and Emotional Health Outcomes among Africans during the COVID-19 Lockdown Period—A Web-based Cross-Sectional Study. Int J Environ Res Public Health. 2021;18:899. 10.3390/ijerph18030899 33494209 PMC7908555

[5] Workneh F Wang D Millogo O Worku A Chukwu A Lankoande B Knowledge and Practice Related to COVID-19 and Mental Health among Adults in Sub-Saharan Africa. Am J Trop Med Hyg. 2021;105:351-62. 10.4269/ajtmh.21-0219 34161301 PMC8437189

[6] Semo BW Frissa SM The Mental Health Impact of the COVID-19 Pandemic: Implications for Sub-Saharan Africa. Psychol Res Behav Manag. 2020;13:713-20. 10.2147/PRBM.S264286 32982500 PMC7508558

[7] World Health Organization. New WHO estimates: Up to 190 000 people could die of COVID-19 in Africa if not controlled. Available: https://www.afro.who.int/news/new-who-estimates-190-000-people-could-die-covid-19-africa-if-not-controlled. Accessed: 18 April 2022.

[8] Johns Hopkins Coronavirus Resource Center. 2022. COVID-19 Map. Available: https://coronavirus.jhu.edu/map.html. Accessed: 18 April 2022.

[9] Tinto B Salinas S Dicko A Kagone TS Traore I de Rekeneire N Spreading of SARS-CoV-2 in West Africa and assessment of risk factors. Epidemiol Infect. 2020;148:e213. 10.1017/S0950268820002149 32921332 PMC7506176

[10] Uzobo E Ayinmoro AD Trapped Between Two Pandemics: Domestic Violence Cases Under COVID-19 Pandemic Lockdown: A Scoping Review. Community Health Equity Res Policy. 2023; 43:319-328. 10.1177/0272684X211022121 34102910 PMC8193047

[11] Saalim K Sakyi KS Fatema-Tuz-Zohra, Morrison E, Owusu P, Dalglish SL, Kanyangarara M. Reported health and social consequences of the COVID-19 pandemic on vulnerable populations and implemented solutions in six West African countries: A media content analysis. PLoS One. 2021;16:e0252890. 10.1371/journal.pone.0252890 34133438 PMC8208543

[12] Kunzler AM Stoffers-Winterling J Stoll M Mancini AL Lehmann S Blessin M Mental health and psychosocial support strategies in highly contagious emerging disease outbreaks of substantial public concern: A systematic scoping review. PLoS One. 2021;16:e0244748. 10.1371/journal.pone.0244748 33534786 PMC7857635

[13] Tol WA Barbui C Galappatti A Silove D Betancourt T Souza R Mental health and psychosocial support in humanitarian settings: linking practice and research. Lancet. 2011;378:1581-91. 10.1016/S0140-6736(11)61094-5 22008428 PMC3985411

[14] Augustinavicius JL Greene MC Lakin DP Tol WA Monitoring and evaluation of mental health and psychosocial support programs in humanitarian settings: a scoping review of terminology and focus. Confl Health. 2018;12:9. 10.1186/s13031-018-0146-0 29560023 PMC5858133

[15] Haroz EE Nguyen AJ Lee CI Tol WA Fine SL Bolton P What works in psychosocial programming in humanitarian contexts in low- and middle-income countries: a systematic review of the evidence. Intervention. 2020;18:15.

[16] Interagency Standing Committee. IASC Reference Group on Mental Health and Psychosocial Support in Emergency Settings. Available: https://interagencystandingcommittee.org/iasc-reference-group-on-mental-health-and-psychosocial-support-in-emergency-settings. Accessed: 18 April 2022.

[17] Department of Health and Human Services. Code of Federal Regulations, Title 45 Public Welfare, Department of Health and Human Services, Part 46 Protection of Human Subjects. Available: https://www.hhs.gov/ohrp/sites/default/files/ohrp/policy/ohrpregulations.pdf Accessed: 12 February 2023.

[18] Akena D Joska J Musisi S Stein DJ Sensitivity and specificity of a visual depression screening instrument among HIV-positive individuals in Uganda, an area with low literacy. AIDS Behav. 2012;16:2399-406. 10.1007/s10461-012-0267-1 22810893

[19] Kohrt BA Jordans MJD Tol WA Luitel NP Maharjan SM Upadhaya N Validation of cross-cultural child mental health and psychosocial research instruments: adapting the Depression Self-Rating Scale and Child PTSD Symptom Scale in Nepal. BMC Psychiatry. 2011;11:127. 10.1186/1471-244X-11-127 21816045 PMC3162495

[20] Stata Statistical Software. Release 17. StataCorp, College Station, Texas, USA, 2021.

[21] Shapiro SS Wilk MB An Analysis of Variance Test for Normality (Complete Samples). Biometrika. 1965;52:591-611. 10.1093/biomet/52.3-4.591

[22] Efron B, Tibshirani R. An introduction to the bootstrap. Boca Raton: Chapman & Hall, 1993.

[23] Shanks L Ariti C Siddiqui MR Pintaldi G Venis S de Jong K Counselling in humanitarian settings: a retrospective analysis of 18 individual-focused non-specialised counselling programmes. Confl Health. 2013;7:19. 10.1186/1752-1505-7-19 24041036 PMC3849884

[24] Aluh DO Onu JU The need for psychosocial support amid COVID-19 crises in Nigeria. Psychol Trauma. 2020;12:557-558. 10.1037/tra0000704 32567871

[25] Tindle R Hemi A Moustafa AA Social support, psychological flexibility and coping mediate the association between COVID-19 related stress exposure and psychological distress. Sci Rep. 2022;12:8688. 10.1038/s41598-022-12262-w 35606392 PMC9126245

[26] Ojokoh BA Makinde OS Fayeun LS Babalola OT Salako KV Adzitey F Impact of COVID-19 and lockdown policies on farming, food security, and agribusiness in West Africa. Data Science for COVID-19. 2022:209-23. 10.1016/B978-0-323-90769-9.00014-1

[27] Anyanwu JC Salami AO The impact of COVID-19 on African economies: An introduction. Afr Dev Rev. 2021;33 Suppl 1:S1-16. 10.1111/1467-8268.12531 34149237 PMC8207010

[28] Interagency Standing Committee. IASC Common Monitoring and Evaluation Framework for Mental Health and Psychosocial Support in Emergency Settings: With means of verification (Version 2.0). Available: https://interagencystandingcommittee.org/iasc-reference-group-mental-health-and-psychosocial-support-emergency-settings/iasc-common-monitoring-and-evaluation-framework-mental-health-and-psychosocial-support-emergency. Accessed: 22 April 2022.

[29] Kohrt BA Kaiser BN Measuring mental health in humanitarian crises: a practitioner’s guide to validity. Confl Health. 2021;15:72. 10.1186/s13031-021-00408-y 34565416 PMC8474916

[30] Habicht JP Victora CG Vaughan JP Evaluation designs for adequacy, plausibility and probability of public health programme performance and impact. Int J Epidemiol. 1999;28:10-8. 10.1093/ije/28.1.10 10195658

